# Foreign Correspondence

**Published:** 1862-08

**Authors:** 


### FOREIGN CORRESPONDENCE.

### EXTRACTS FROM A LETTER FROM E. L. HOLMES, M. D.

VIENNA, July 15, 1862.

Probably few medical students or even practicing physicians, who contemplate visiting the European hospitals for the purpose of professional study, form a correct idea either of the advantages which they may really enjoy, or of the peculiar difficulties which they will encounter.

It was my good fortune, seven or eight years since, to spend eighteen months in Europe, visiting principally the hospitals of Paris and Vienna; and I am now revisiting the same hospitals for a few months, partly for pleasure and recreation and partly for professional improvement. Possibly the experience of my former residence here, together with an experience of seven years of quite active practice, may now enable me to offer the readers of the JOURNAL an intelligent statement of the advantages and disadvantages which the members of our profession will usually meet while pursuing their studies in the French or German hospitals.

The time is past when the mere fact that a young man had "followed the hospitals" of Paris or London, was alone sufficient to establish his reputation as a physician, in almost any city in which he might commence practice. A visit to Europe was then regarded of so much importance, because at that period in the history of medical science in America, the whole country offered scarcely any opportunity for the student to investigate disease, except as described in books. To do this he was obliged to visit Europe, whose hospitals presented so many examples of every form of disease. Fortunately, during the last quarter of a century, the facilities in our large cities for the study of disease at the bed-side, have become very great, and the time is undoubtedly not far distant when these facilities will be all the student can desire. Our own city of Chicago may already be justly proud of the reputation of its medical teachers, and the friends of professional culture in the Northwest may be encouraged at the increasing opportunities for clinical observation, which the student may now enjoy, and yet there are certain benefits connected with a course of medical study in Europe, which will for years render it desirable for every physician, if possible, to spend a year or two in clinical observation abroad.

There are perhaps two great advantages which the student secures in the large European cities. In the first place the number of patients in the hospitals of these cities is almost incredibly large, and furnish an unlimited amount of material, from which numerous examples of every form of disease may be presented to the observation of the student. The second advantage which the American student will find, depends upon the fact, that here, more than in the United States, there are a larger number of instructors in special diseases, who have for years been in the habit of teaching at the bed-side. There are in the United States many of our own profession whose knowledge, perhaps, in any special department of medicine, is sufficient for this. In addition to knowledge, the successful clinical teacher must possess a faculty of imparting his knowledge, which can usually be acquired by practice alone.

In Europe there is no prejudice againt specialists as in the United States; the most distinguished men are those who have made special classes of disease the study of their lives, and are eminently capable of teaching in their respective branches. There are certain reasons why specialists in the United States have not received the confidence and encouragement of the profession at large. Unfortunately with us, so called specialists are almost always not only ignorant of medicine in general, but also of the particular class of diseases in which they pretend to have acquired peculiar skill in treatment. Medical science is already too broad for any mind to attempt to grasp all that is known in its various departments. The physician who has acquired a knowledge of the general principles of medicine, and who then devotes himself to the study and treatment of one class of diseases will certainly excel, in his particular branch, others who devote their time and energies to the whole range of professional labor. We need but turn to the history of the progress of medical science, to be assured that our profession is indebted almost wholly to specialists, for every great discovery in the science, and for every great work in the literature of medicine. And they who have become most distinguished in Pathology, Anatomy, Physiology, and who have acquired a world wide reputation for their skill in the diagnosis and treatment of the chest, of the eye or ear, of the nerves, or of any special organ, have been those who have made the study of comparatively a few medical subjects the object and aim of life. And, notwithstanding the existing prejudice, the works of these specialists are received as authority, by those even who are most disposed to discountenance a similar division of labor in our own country. Undoubtedly in a few years this division of labor will be regarded with as much favor by the profession generally in our own country as in Europe.

Briefly then, the great advantages which the student will enjoy in some of the European cities, are these :—He will make the acquaintance and receive the instruction of physicians who, if possible, not only know all that human skill and ingenuity has as yet brought to light in their respective departments of medical science, but are also through long experience, accustomed to lecture at the bed side. Moreover, they have at their disposal a very large number of cases to illustrate every point connected with each disease.

The principal difficulty which most American students will meet in pursuing their studies in Europe, consists in the fact that the instruction is given in a foreign language, and every student who is without previous discipline in the study of language, should know that generally it is no small task to acquire a sufficient knowledge of either French or German to enable him to follow the ordinary clinical lectures. Few young men can do this in less than six months. I am fully convinced that most students who are unacquainted with either of these languages, and who intend spending but a year in Europe, will derive much more benefit in devoting their time and money to study in the hospitals of Great Britain or in those of our Eastern cities. In my next letter I will describe in detail the splendid hospitals of this city, its numerous churches, its splendid Pathological museum, its large number of private courses of instruction, and enumerate the peculiar advantages which Vienna offers the student more than any other city.

